# Modeling the allosteric modulation on a G-Protein Coupled Receptor: the case of M2 muscarinic Acetylcholine Receptor in complex with LY211960

**DOI:** 10.1038/s41598-020-59289-5

**Published:** 2020-02-20

**Authors:** L. Maggi, P. Carloni, G. Rossetti

**Affiliations:** 1 Computational Biomedicine Section, Institute of Advanced Simulation IAS-5 and Institute of Neuroscience and Medicine INM-9, Forschungszentrum Jülich GmbH, 52425 Jülich, Germany; 2 Institute for Neuroscience and Medicine INM-11, Forschungszentrum Jülich, 52428 Jülich, Germany; 3 Department of Physics, RWTH Aachen University, 52078 Aachen, Germany; 4 Department of Neurology, University Hospital Aachen, 52078 Aachen, Germany; 5 Simulation Laboratory Biology, Jülich Supercomputing Centre (JSC), Forschungszentrum Jülich GmbH, 52428 Jülich, Germany; 6 Department of Oncology, Hematology, Oncology, Hemostaseology, and Stem Cell Transplantation, University Hospital Aachen, RWTH Aachen University, 52074 Aachen, Germany

**Keywords:** Computational biophysics, Drug discovery

## Abstract

Allosteric modulation is involved in a plethora of diverse protein functions, which are fundamental for cells’ life. This phenomenon can be thought as *communication* between two topographically distinct site of a protein structure. How this communication occurs is still matter of debate. Many different descriptions have been presented so far. Here we consider a specific case where any significant conformational change is involved upon allosteric modulator binding and the phenomenon is depicted as a vibrational energy diffusion process between distant protein regions. We applied this model, by employing computational tools, to the human muscarinic receptor M2, a transmembrane protein G-protein coupled receptor known to undergo allosteric modulation whose recently X-ray structure has been recently resolved both with and without the presence of a particular allosteric modulator. Our calculations, performed on these two receptor structures, suggest that for this case the allosteric modulator modifies the energy current between functionally relevant regions of the protein; this allows to identify the main residues responsible for this modulation. These results contribute to shed light on the molecular basis of allosteric modulation and may help design new allosteric ligands.

## Introduction

Allosteric modulation of proteins, discovered more than fifty years ago^1^, plays an important role for many processes, from signal transduction^2^ to transcriptional regulations^3^.

This phenomenon regards any molecular event in which the binding of a ligand in a specific region of a protein (*allosteric binding site*) affects the stability of distant “primary” binding site (often referred as *orthosteric binding site)*^4,5^. The term allosteric is coined for the first time by Monod, Wyman and Changeux within the WMC model^6^. According to it, proteins can assume two different conformations, each of them exhibiting different binding affinity for the orthosteric ligand. The allosteric ligands can affect the thermodynamic stability of this conformations modifying, consequently, the orthosteric binding affinity. This model implies a protein conformational change mediating the interaction between the two distant sites. However, it has been shown that the allosteric binding site can affect the orthosteric binding also without involving any conformational rearrangement^7^, modifying, for instance, proteins vibrations around their thermodynamic stable conformations^7^. These different types of allosteric modulation share a common and peculiar feature, namely the presence of a *long-range communication* between two different sites of a protein^8^. Allosteric modulators can be distinguished on the basis of their contribution to the free energy of binding. In the case that such contribution is manly enthalpic, (Type I), it usually leads to a conformational change^9^. In contrast, when entropic contribution is prevalent (Type II), molecular vibrations are mainly affected. Usually then, no appreciable conformational changes are observed^9^. In this work we will consider only this case. Finally, cases in between the first two types are also possible^9^. Allostery can be exploited for drug development. Indeed, allosteric ligands can cause an increase the orthosteric ligands’ affinity for their protein target^10^. These ligands (“Positive Allosteric Modulators”, PAMs) turn out to decrease the dissociation constant (*K*_*d*_) of the orthosteric ligand upon target binding. In contrast, Negative Allosteric Modulators (NAMs) increases *K*_*d*_ while Neutral Allosteric Ligand (NALs) do not affect *K*_*d*_^11^. Understanding the molecular basis of allosteric modulation and identifying the key elements which make this phenomenon possible is therefore of great importance for both basic science and drug discovery.

**Figure 1:**
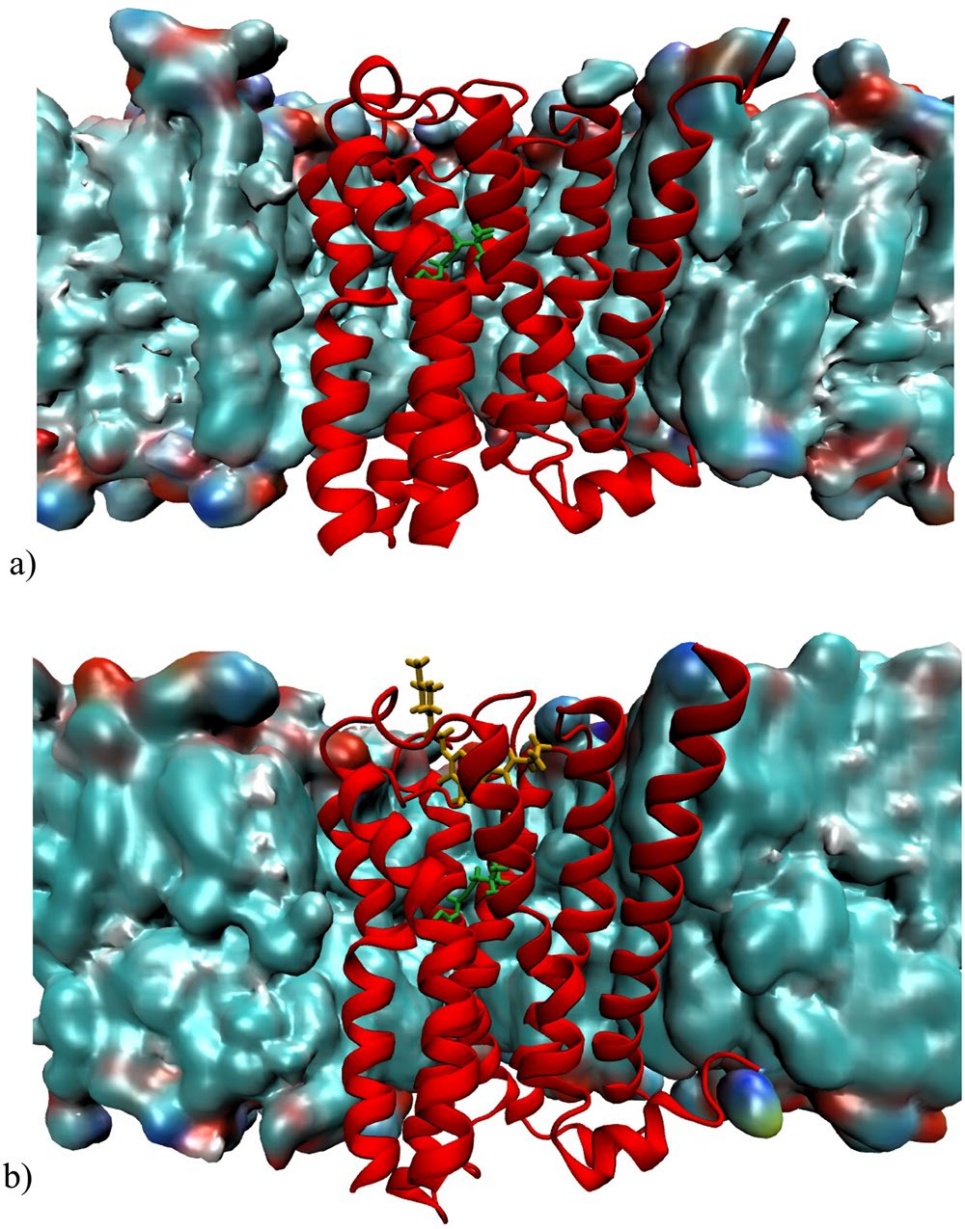
M2 X-ray Crystal Structures. (**a**) M2 muscarinic acethylcholine receptor bound to an orthosteric agonist (Iperoxo), in green. (**b**) M2 muscarinic acethilcholyne receptor bound to the allosteric modulator (LY211960), in orange, and the agonist. Both complexes are embedded in a membrane (See methods). These figures have been created with VMD 1.9.3 software package^39^. (http://www.ks.uiuc.edu/Research/vmd/).

We use as a test case the M2 muscarinic acetylcholine receptor (M2), for which large structural information are available. This receptor belongs to the class A of the G-protein Coupled receptor (GPCRs) superfamily. GPCRs are transmembrane proteins which trigger a signal cascade inside the cell upon binding with the G-protein. The allosteric binding occurs prevalently when the receptor assumes a particular conformation called *active state*^12^. In most cases, the active state is promoted, by the binding of small molecules called agonist^13^. Here we study the binary complex between M2 with its agonist iperoxo as well as the ternary complex between the receptor, iperoxo and the PAM LY211960, whose X-ray structures have been determined (Fig. 1a,b).

GPCRs possess very complex energy landscape presenting several stable conformations^14^. Allosteric modulators can modify this landscape affecting the stability and/or the dynamical properties of each conformation^8^. In this respect, our study focuses on two stable states of a GPCR/ligand adduct. The two states are expected to be two free energy minima. It reasonable to assume that they are active states^15^. The two X-ray structures are not too dissimilar, indeed, their RMSDs differ by only about 1 Å and this difference remains small during a molecular dynamic simulation (see Fig. SI2). Therefore, the allosteric modulation, in this particular case, does not seem to involve a significant shift in the population of conformational states. Rather, it causes a local reshape of the energy landscape of the same conformational state^16^. Investigating cases in which the allosteric modulation leads to a population shift in the conformational states is beyond the scope of this paper.

Therefore, in this case, it is reasonable to think the allosteric modulation occurs by means of molecular vibrations of the protein involving the transfer of the associated energy, the vibrational energy (*E*_*vib*_). In the following, the allosteric modulation will be indeed modeled as exchange of *E*_*vib*_ among protein residue. This study will be focused only on this specific Type II PAM since sufficient experimental data and studies are available^15,17,18^.

## Results and Discussion

As stated above, in this work we describe the allosteric modulation as *communication* which consist in the *E*_*vib*_ exchange among protein residues. This exchange is modeled as a diffusion process to which a master equation can be associated with.

1 $$\frac{d{\boldsymbol{p}}(t)}{dt}={\boldsymbol{p}}(t)\cdot {\boldsymbol{L}}$$

***p***(*t*) is a N-entries vector, where N is the number of residues. Each of its entries represents the percentage of total *E*_*vib*_ relative to each residue at time *t* (e.g. ${p}_{i}(t)=\frac{{E}_{vib}^{res\,i}(t)}{{E}_{vib}\,}$, where ${E}_{vib}^{res\,i}(t)$ is the vibrational energy of the *i*-th residue. Obviously, we do not the exact value of ${E}_{vib}^{res\,i}(t)$ and *E*_*vib*_ but just their ratio *p*_*i*_(*t*)). ***L*** is the transition frequency matrix, whose elements ***L***_***ij***_ are the frequencies associated with transition times ***τ***^***ij***^, which are the characteristic time to exchange *E*_*vib*_ between residue *i* and *j*, as calculated in reference^19^. Each element of ***L*** is defined as:

2 $${{\boldsymbol{L}}}_{ij}=\{\begin{array}{c}\frac{1}{\,{\tau }^{ij}\,}\,if\,i\ne j\,\\ -\sum _{k\ne i}\frac{1}{\,{\tau }^{ik}\,}\,if\,i=j\end{array}$$

The Master Eq. (1) defines a Transition Network (Markov State Model or simply Markov chain) in which nodes are residues connected via edges whose weights are the element (***L***_***ij***_) of the matrix. ***L*** can be decomposed in its eigenvectors. Each of them is associated to an eigenvalue, i.e. a frequency. The eigenvalue zero represents the thermal equilibrium, in which all the residues have the same amount of *E*_*vib*_, and the corresponding eigenvectors (***p***^∞^) is the so-called stationary distribution since *d****p***^∞^/*dt* = ***p***^∞^·***L*** = 0.

All the other eigenvectors represent metastable states, through which the system pass by to reach the thermal equilibrium and they “exist” for a finite period of time. In other words, they describe states in which some residues possess a larger *E*_*vib*_ than others. This difference causes the energy exchange that we decided to employ for modeling the allosteric communication. Therefore, identifying group of residues which can retain more *E*_*vib*_ during the process of reaching the equilibrium can be highly interesting because they might represent biologically relevant regions and studying *E*_*vib*_ exchange among them will shed light on how allosteric modulation occurs in protein. Therefore, in the following, we will identify first this regions and study how the *E*_*vib*_ exchange occurs among them.

Our analysis focuses here on all the residues of the M2 (Fig. 1), except those belonging to the third intracellular loop. Due to its high mobility, indeed, the approximations needed for calculating *τ*^*ij*^ no longer holds^19^ and all the possible values becomes unreliable. The loop is included in our MD simulations (see Methods). For the β2 adrenergic (another class A GPCR such as the M2 receptor), this loop is a distinct domain not affecting the seven helices bundle structural features^20^. One can reasonably expect the same for the M2 receptor, because of the structural similarity of the two receptors.

Here, we consider both the binary (receptor/iperoxo) complex with the agonist iperoxo and its the ternary complex, where also the allosteric ligand LY211960 is present.

**Figure 2:**
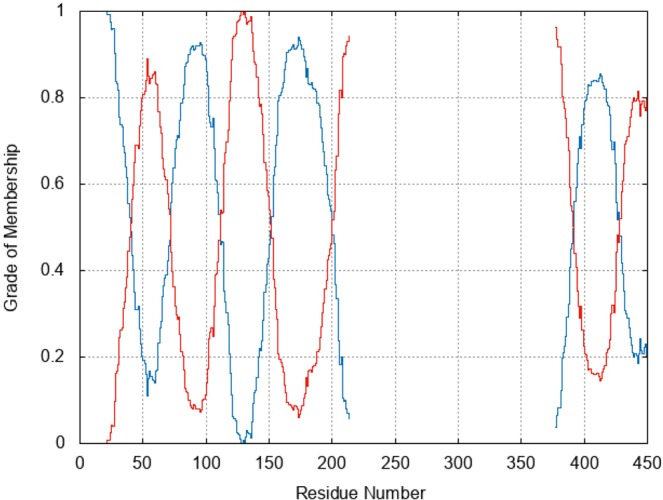
Clusters Grade of membership. Grade of membership for the two identified residue clusters, distinguished by the color of the lines.

**Figure 3:**
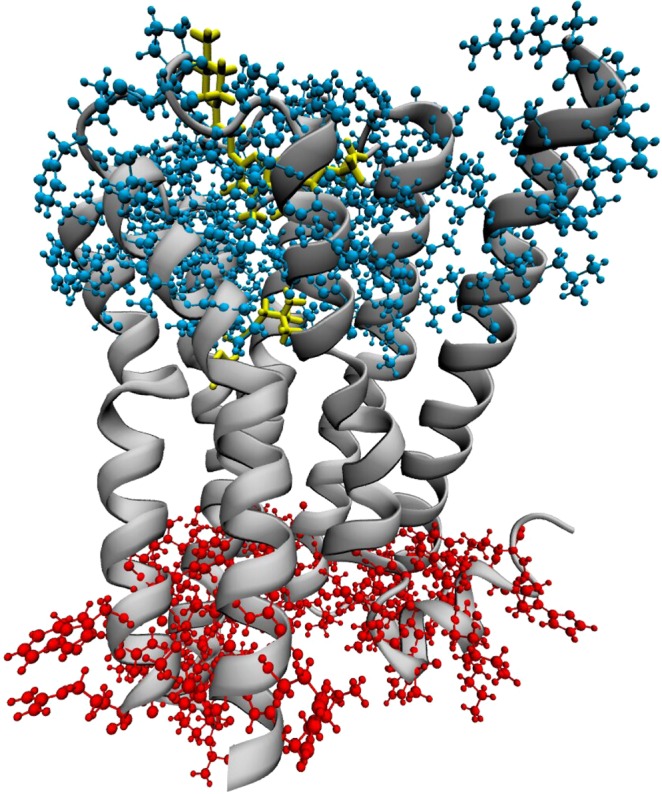
The two identified clusters. The orthosteric ligand depicted in yellow for showing the location of the two binding sites with respect to the clusters. This figure has been created with VMD 1.9.3 software package^39^. (http://www.ks.uiuc.edu/Research/vmd/).

We first used the PCCA+^21^ approach to identify clusters of the residues related to metastable states. This requires to set the number of clusters (*n*), using a ‘goodness’ criterion: A specific parameter, *θ*, which depends on the number of clusters *n*, should be as close to zero (optimal value) as possible. It turns out that setting *n* = 2 leads to *θ* = 0, while is much smaller 0 for *n* > 2 (See methods). Therefore, setting *n* = 2, protein residues are distributed among two clusters and each of them can be assigned to one cluster calculating the “grades of membership” for each residue. This ranges from 0, when the residue does not belong to the specific cluster, to 1, when the residue belongs only to the cluster (Fig. 2). Here, we establish a cutoff beyond which the residue is assigned to a specific cluster. If the cutoff is set to 0.7, the two resulting clusters turn out to include residues crucial for the binding of the cognate G-proteins^12^ as well as the allosteric and orthosteric ligands^15,22^ (see Fig. 3). These clusters have been identified both in the binary and the tertiary complex. Choosing a higher cutoff such as 0.8, 0.9 turns out not to significantly affect our results (data not shown).

Those residues playing a role in allosteric or orthosteric binding might be instrumental for drug development, therefore, their relevance to *E*_*vib*_ exchange is here investigated. According to PCCA+, the two recognized clusters represents regions in which the *E*_*vib*_ exchange processes among residues within the cluster is faster than the *E*_*vib*_ exchange between the clusters themselves. This finding agrees with previously reported computational results showing that residues belonging to the regions identified by the clusters feature shorter characteristic time for *E*_*vib*_ exchange^19^.

**Figure 4:**
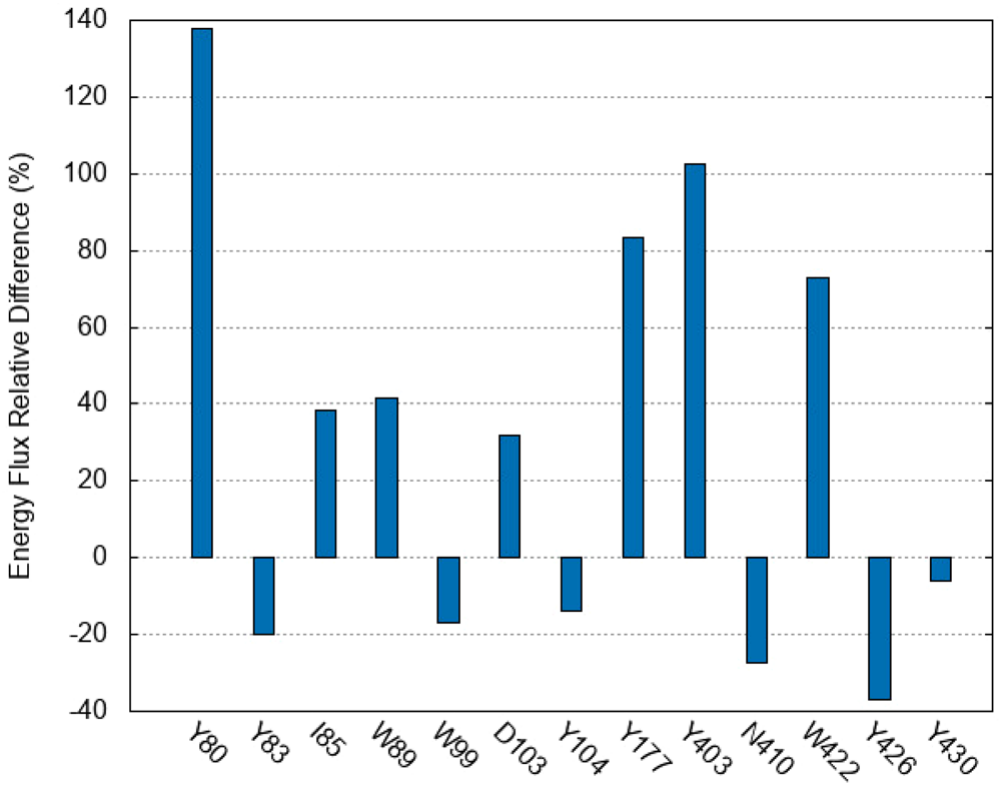
Relevant Residues for the allosteric Modulation. Side (**a**) and top (**b**) views of the receptor showing the residues considered in our analysis and the allosteric and orthosteric ligand. They are mostly aromatic residues either binding directly the allosteric modulator (Y80, Y83, I85, W89, W99, Y177, N410, W422) or the orthosteric (D103, Y104.Y403, Y426, Y430).

**Figure 5:**
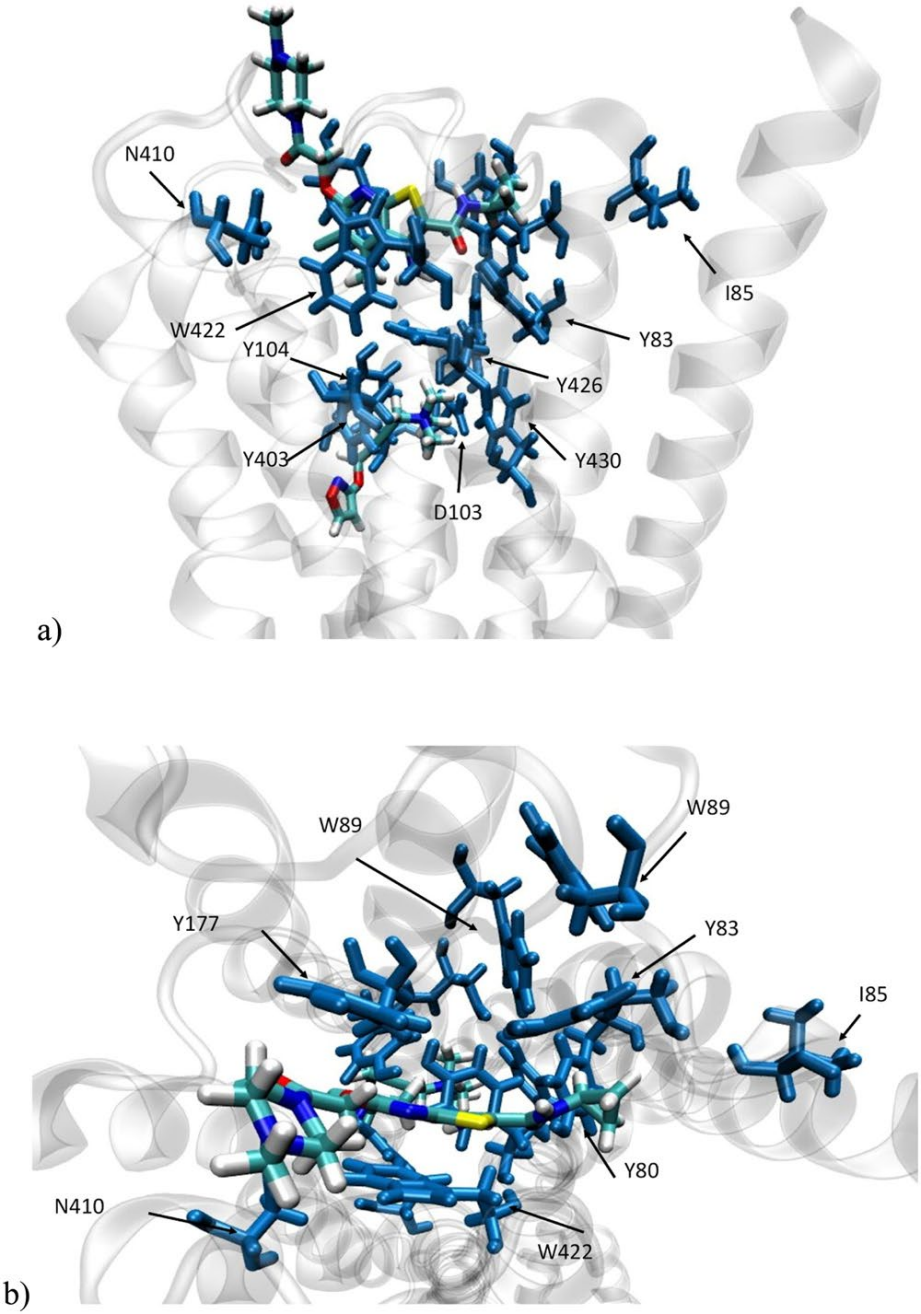
Contribution to energy current. Energy current of considered residues difference between the allosteric and orthosteric ligand bound complexes. These figures have been created with VMD 1.9.3 software package^39^. (http://www.ks.uiuc.edu/Research/vmd/).

The energy current *F* between the two clusters, calculated using the so-called Transition Path Theory (TPT) (see Methods), increases on passing from the binary (*F*_*ag*_ hereafter) to the ternary complex (*F*_*all*_). The relative percentage change (Δ*F*) between the two complexes, which reads:

3 $$\Delta F=\frac{{F}_{all}-{F}_{ag}}{{F}_{ag}}\cdot 100$$

is around 20%. Thus, in our specific complex, the presence of the allosteric ligand causes a speeding up of the energy exchange between distant parts of the protein. This might play a biologically relevant role, as it affects the *communication* among two distant biologically active sites. On the other hand, it might make the entire structure more “stable”, since any thermal perturbation can be accommodated faster among the degrees of freedom. This allows the protein to faster reach the thermal equilibrium. It should be noticed that this finding strictly holds for the case considered, although It is plausible (albeit not proved) that other GPCRs/allosteric ligands sharing a high degree of structural similarity with the one considered here might exhibit similar behavior binding an allosteric modulator belonging to the same class as LY211960. Conversely, what makes this study specific for the M2 receptor is the analysis of the residues involved in allosteric and orthosteric binding. This is carried out by calculating Δ*F*_*X*_ for a specific residue *X*, as opposed to the overall Δ*F* value in Eq. (3). The question we will address is: Does a large value of Δ*F*_*X*_ indicate an important role of the residue for ligands binding? Fig. 4 shows that the largest Δ*F*_*X*_ values (>70%) are those for X = Y80, W422, Y177 and Y403. Interestingly, all of these residues are important for binding (see Fig. 5) (i) W422 and Y177 play a crucial role for the binding itself^22^. They form two pi-stacking bonds on two opposite sides of the ligand aromatic rings. Binding does not occur if they are mutated with a non-aromatic residue^22^. (ii) Y403, which interacts with W422 through a T-shaped stacking, is part of tyrosine lid that has been shown to be essential for the orthosteric binding^23,24^. (iii)Y80’s hydroxyl group forms a stable hydrogen bond with the allosteric ligand^15^, pointing to his role for binding. The latter is further confirmed by the change in *magnitude of positive cooperativity* (Δ*α*) upon mutation of the residue to alanine in the structurally similar M4 receptor^22^. ^a^^1^*α* is the ratio between the dissociation constant of the orthosteric ligand with and without the allosteric modulator: it can be considered as the relative difference in the *E*_*vib*_ current and a measure of the relative loss/gain of stability upon binding of the allosteric modulator. Δ*α* value is the largest so far among all those measured (Fig. SI1). The relevance these residues have, both for the *E*_*vib*_ exchange and for the allosteric/orthosteric binding, makes them useful for being exploited for drug development. Noteworthy the interactions they make with the allosteric ligands modifies their dynamics and, thus, their property of exchanging *E*_*vib*_ with other residues. Even a slightly change in those interactions might lead, consequently, to either a decrease or a negligible value of Δ*F* producing a different allosteric behavior.

## Conclusion

We have presented a molecular simulation study on the M2 receptor in complex with its agonist and the PAM LY211960. Our calculations show that the latter affects the dynamics and consequently the *E*_*vib*_ exchange in the M2 receptor structure. Upon LY211960 binding, the energy between distant protein regions is speeded up. Thus, any kind of perturbation affecting the structure could be accommodated easier than in the case the ligand is absent. Residues involved in either allosteric or orthosteric binding, showed to affect the energy current change on passing from the binary to the ternary complex. The last finding may provide valuable information for further allosteric drug development. Indeed, these residues can be exploited to model new ligands targeting specifically them and effectively modifying the allosteric communication. These results might be generalized to other proteins accommodating PAMs.

## Methods

### Systems and calculations

The calculations are based on the mAchR iperoxo binary (PDBID 4MQS) and mAchR iperoxo LY211960 (PDBID: 4MQT) ternary complex X-ray structures. A long intracellular loop between residues 212 to 383, not present in the crystal structures, was added using the Robetta^25^ and MODELLER^26^ codes.

The proteins were inserted in a pre-equilibrated membrane, built using CHARMM-GUI membrane builder web server^27^. 80.000 water molecules were added. Na^+^ and Cl^−^ ions were also added so as to make the system neutral and to reach a physiological concentration (about 0.15 M). The overall systems consisted of about 120.000 atoms.

The AMBER99SB^28^ force field and TIP3P model^29^, Slipids^30^ were used for the protein, Na^+^ and Cl^−^ ions, water and lipids. The General Amber force field (GAFF) parameters^31^ were used for the ligands in both complexes, along with the RESP atomic charges^32^. These were obtained by fitting with the electrostatic potential (ESP) from Gaussian 09^33^ calculation with the HF-6-31G* basis set. The ligands topologies were converted to the GROMACS format using the ACPYPE tool^34^.

One simulation for each system was performed with the GROMACS program suite^35^. 500 ns were performed in the NPT ensemble using 1 fs time-step, sampling each 100 ps, after a 50 ns of thermalization run, in a 11 × 11 × 20 A simulation box. Nose-Hoover thermostat^36^ was used with τ = 0.4 ps along with Parrinello-Rahman barostat^37^ with τ = 1.0 ps. Electrostatic interactions were treated using PME method^38^. A cut-off of 1.2 nm was used for all the long-range interactions.

### Robust perron cluster ananlysis

PCCA+^21^ aims to identify metastable states associated clusters to find a diagonal-block structure in the *N* by *N* transition frequency matrix ***L***. To do so, an arbitrary number *q* of excepted clusters is defined. The “goodness” of this choice can be tested *a posteriori* using an estimator, *θ*.

C_k_⊂{1, … *N*} is the set of indices (of the matrix ***L***) of the *k*-th cluster, *k* = 1, … *q*.

Furthermore, the set of representative indices *π*(*k*) of the *i*-th cluster is defined so that a row **t**_*π*(*k*)_ of ***L*** is orthogonal to the characteristic vector of the cluster *l* (${{\boldsymbol{\chi }}}_{l}$):

4 $${{\boldsymbol{t}}}_{\pi (k)}\cdot {{\boldsymbol{\chi }}}_{{C}_{l}}={\delta }_{kl}$$

where *δ*_*kl*_ is the Kronecker delta. ${{\boldsymbol{\chi }}}_{{C}_{l}}$ is zero if the index of the entries does not belong to *C*_*l*_. For a generic row ***t***_*m*_ the Eq. (4) becomes:

5 $${{\boldsymbol{t}}}_{m}\cdot {{\boldsymbol{\chi }}}_{{C}_{k}}={p}_{m,{C}_{k}}$$

where ${{p}}_{{m}{,}{{C}}_{{i}}}$ is the transition probability from the state *m* to the cluster *C*_*i*_. Hence, combining Eqs. (4) and (5) we get:

6 $$({{\boldsymbol{t}}}_{m}-\mathop{\sum }\limits_{s=1}^{q}{p}_{m,{C}_{s}}{{\boldsymbol{t}}}_{\pi (s)})\cdot {{\boldsymbol{\chi }}}_{{C}_{k}}=0$$

This means the error one commits writing ***t***_*m*_ as a linear combination **t**_*π*(*s*)_ is orthogonal to every ${{\boldsymbol{\chi }}}_{{C}_{k}}$. In principle we don’t know ${{\boldsymbol{\chi }}}_{{C}_{k}}$ but we know that, if there is a hidden block-diagonal structure, the ***L***’s eigenvector *e*^(*k*)^ are almost constant on *C*_*k*_. This means that we can approximate the Eq. (6) as:

7 $$({{\boldsymbol{t}}}_{m}-\mathop{\sum }\limits_{s=1}^{q}{w}_{m}^{(s)}\,{{\boldsymbol{t}}}_{\pi (s)})\cdot {{\boldsymbol{e}}}^{(k)}=0$$

where we have introduced ${w}_{m}^{(s)} \sim {p}_{m,{C}_{s}}$, which are defined as the grade of membership of the state *m* to the cluster *s* and they should range from 0 to 1. *w*_*m,s*_ is the main outcome of PCCA+. In order to calculate them we can recast Eq. (7) as:

8 $${{\boldsymbol{e}}}_{m}^{(k)}=\mathop{\sum }\limits_{s=1}^{q}{w}_{m}^{(s)}{{\boldsymbol{e}}}_{\pi (s)}^{(k)}$$

Knowing *π*(*s*) and the eigenvectors *e*^(*k*)^ the Eq. (8) can be inverted for finding ${w}_{m}^{(s)}$. *e*^(*k*)^ are obtained by diagonalizing ***L*** and *π*(*s*) are chosen selecting those which maximize the distance ||***e***_*r*_−***e***_*p*_|| where *e*_*r*(*p*)_ are vectors in ${{\mathbb{R}}}^{q}$ so that *e*_*r*(*p*)_ = (${{\boldsymbol{e}}}_{r(p)}^{(1)},\ldots ,{{{\boldsymbol{e}}}_{r(p)}}^{(q)}$)^21^.

Finally, we have to discuss the chosen number of clusters. Ad stated above ${w}_{m}^{(s)} \sim {p}_{m,{C}_{s}}$, hence, ${w}_{m}^{(s)}\ge 0$. There is no guarantee the latter is fulfilled, therefore, the positiveness of ${w}_{m}^{(s)}$ could be used to evaluate if the number of chosen clusters *q* is suitable for the system under consideration or not. Thus, we define *θ* as:

9 $$\theta =mi{n}_{k,m}{w}_{m}^{(k)}$$

whereas $\theta \ll 0$ the number of clusters is too large.

### Transition path theory

Given two sets of residues A and B (Identified by PCCA+), we define a trajectory on the Markov chain as a sequence of residues over time. The probability that a trajectory starting from a residue *i* ∈(*A*∪*B*)^c^ reaches first A then B is called *forward committor probability* ${q}_{i}^{+}$ and it solves the following set of equations:

10 $$\begin{array}{cc}\sum _{j}{{\boldsymbol{L}}}_{ij}{q}_{j}^{+}=0 & \forall \,i\in {(A\cup B)}^{c}\\ {q}_{i}^{+}=0 & \forall \,i\in A\\ {q}_{i}^{+}=1 & \forall \,{\rm{i}}\in B\end{array}$$

Conversely, we can define the *backward committor probability* ${q}_{i}^{-}$, which is the probability of reaching *i* first than B starting from A and, as in the case of ${q}_{i}^{+}$, the following equations hold :

11 $$\begin{array}{cc}\sum _{j}{{\boldsymbol{L}}}_{ij}{q}_{j}^{-}=0 & \forall \,i\in {(A\cup B)}^{c}\\ {q}_{i}^{-}=1 & \forall \,i\in A\\ {q}_{i}^{+}=0 & \forall \,i\in B\end{array}$$

Trajectories going from A to B are called *reactive trajectories*. The probability to find *reactive trajectories*, given *i* ∈ (*A*∪*B*)^c^ is:

12 $${m}_{i}^{R}={p}_{i}^{\infty }{q}_{i}^{-}{q}_{i}^{+}$$

where ${p}_{\alpha }^{\infty }$ is the stationary probability distribution. Finally, we can define the probability current of reactive trajectories “flowing” from *i* to *j*, with *i, j* ∈ (*A*∪*B*)^c^ and *i* ≠ *j* as:

13 $${f}_{ij}^{AB}={p}_{i}^{\infty }{q}_{j}^{-}{{\boldsymbol{L}}}_{ij}{q}_{j}^{+}$$

In conclusion, the probability current exiting from a cluster A and entering in B is :

14 $${F}^{AB}=\sum _{\alpha \in A}\sum _{\beta \notin A}{f}_{ij}^{AB}$$

This current is directly connected to the *E*_*vib*_ current by a multiplicative constant. However, in this work we are interested in relative changes in the flux and not in their absolute values, thus, our results hold independently this constant.

## Supplementary information

Supplementary information.

## Footnotes

1 ^a^M4 belongs to the same subclass of GPCRs as M2, it binds the LY211960 allosteric ligand with the same affinity as M2^15^ and it shares 75% sequence identity with M2, as calculated with BLAST web server^24^25. Altschul, S. F., Gish, W., Miller, W., Myers, E. W. & Lipman, D. J. Basic local alignment search tool. *J Mol Biol* **215**, 403–410 (1990).
