# Supplementary material for: Modeling the allosteric modulation on a G-Protein Coupled Receptor: the case of M2 muscarinic Acetylcholine Receptor in complex with LY211960
Source: Sci Rep. 2020 Feb 20;10:3037. doi: 10.1038/s41598-020-59289-5 (PMC7033091; doi:10.1038/s41598-020-59289-5)
Supplement: Supplementary file 1 — Supplementary information. [file 41598_2020_59289_MOESM1_ESM.docx]

### Modeling the allosteric modulation on a G-Protein Coupled Receptor: the case of M2 muscarinic Acetylcholine Receptor in complex with LY211960

**(Suppoting Information)**

L. Maggi^a *^, P. Carloni^a,b,c^ ,G. Rossetti ^a,d,e,f^

^a^ Computational Biomedicine Section, Institute of Advanced Simulation IAS-5 and Institute of Neuroscience and Medicine INM-9, Forschungszentrum Jülich GmbH, 52425 Jülich, Germany

^b^ Institute for Neuroscience and Medicine INM-11, Forschungszentrum Jülich, 52428 Jülich, Germany

^c^ Department of Physics, RWTH Aachen University, 52078 Aachen, Germany

^d^ Department of Neurology, University Hospital Aachen, 52078 Aachen, Germany

^e^Division Computational Science − Simulation Laboratory Biology, Jülich Supercomputing Centre (JSC), Forschungszentrum Jülich GmbH, 52428 Jülich, Germany

^f^Department of Oncology, Hematology and Stem Cell Transplantation, University Hospital Aachen, RWTH Aachen University, 52074 Aachen, Germany

L.M. is the corresponding author: [*l.maggi@fz-juelich.de](mailto:*l.maggi@fz-juelich.de)


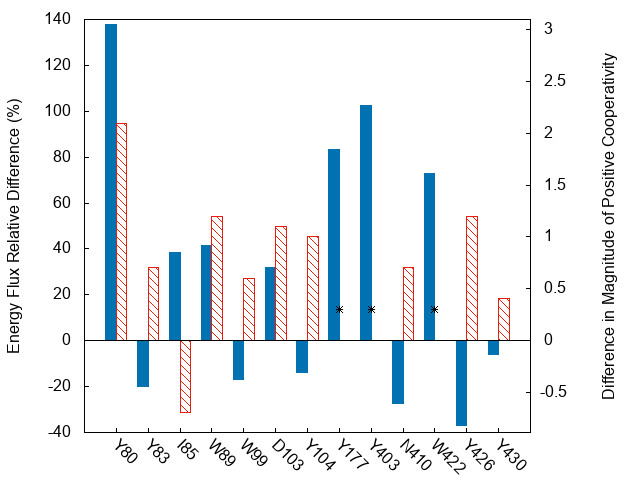


**Figure SI 1**:Contribution to energy current. Energy current of considered residues difference between the allosteric and orthosteric ligand bound complexes (blue bars) and the difference in the magnitude of positive cooperative (red bars) from Ref. 20 (see main text). The asterisks indicates residues for which this quantity has not been measured.


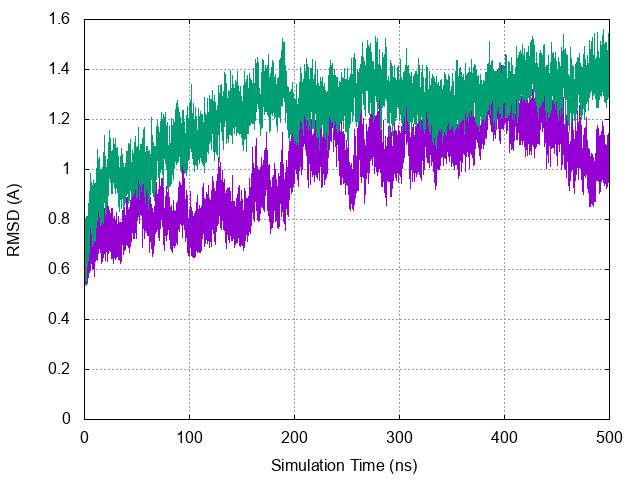


**Figure SI 2**  RMSD plot during 500 ns simulation for the M2 receptor with(green) and without (purple) the allosteric modulator. Those are calculated using the X-ray structures as reference
